# Targeted-gene sequencing of an undifferentiated gallbladder carcinoma: a case report

**DOI:** 10.1186/s13000-020-00981-5

**Published:** 2020-06-02

**Authors:** Ying Xiao, Canhong Xiang, Di Yang, Benqi Zhao, Yong Li, Hongfang Yin

**Affiliations:** 1 Department of Pathology, Beijing Tsinghua Changgung Hospital, School of Clinical Medicine, Tsinghua University, Beijing, PR China; 2 Department of Hepatopancreatobiliary Surgery, Beijing Tsinghua Changgung Hospital, School of Clinical Medicine, Tsinghua University, Beijing, PR China; 3 Department of Radiology, Beijing Tsinghua Changgung Hospital, School of Clinical Medicine, Tsinghua University, Beijing, PR China

**Keywords:** Undifferentiated carcinoma, Gallbladder carcinoma, Case report, Targeted gene sequencing, Tumor mutation burden

## Abstract

**Background:**

Undifferentiated carcinomas of the gallbladder are extremely rare. Most undifferentiated carcinomas are accompanied by adjacent foci of other conventional carcinomas, and a transition zone is shared between them. However, genetic alterations of undifferentiated gallbladder carcinoma and the similarities or differences between the undifferentiated carcinoma and the foci conventional carcinoma are unknown.

**Case presentation:**

Herein, we report a case of undifferentiated gallbladder carcinoma with osteoclast-like giant cells with invasion into the liver, duodenum, and stomach in a 56-year-old man. The tumor was microscopically formed from the tubular adenocarcinoma (< 5% of the entire tumor), the undifferentiated carcinoma, and a transition zone between them. Four somatic mutations (*TP53, TERT, ARID2,* and *CDH1*), three amplifications (*CCND1, FGF19,* and *MET*), and a tumor mutation burden (TMB) of 3.45 muts/Mb were detected in the undifferentiated component using targeted gene sequencing, whereas 102 somatic mutations (including *TP53, TERT, ARID2,* and *CDH1*), one amplification (*CCND1*), and a higher TMB of 87.07 muts/Mb were detected in the tubular component. This patient died of tumor recurrence 2 months after the surgery.

**Conclusions:**

The undifferentiated gallbladder carcinoma had its unique molecular alterations. The similarities in the genetic alterations of the undifferentiated carcinoma and adenocarcinoma provide evidence of a common origin at the genetic level. The occurrence of an undifferentiated carcinoma may be due to heterogeneity-associated branched evolution from the tubular adenocarcinoma.

## Introduction

An undifferentiated gallbladder carcinoma is a rare epithelial type of cancer with an extremely poor prognosis. It is composed of spindle or giant cells with a diffuse growth pattern and without a definitive direction of differentiation. Owing to its rarity, only a few cases have been reported. Most reported undifferentiated carcinomas are accompanied by the foci of conventional carcinoma, including adenocarcinoma, squamous carcinoma, and adenosquamous carcinoma, having shared transition zones between the foci and the undifferentiated carcinoma portion [1–3]. These transition zones suggest that undifferentiated carcinomas may originate from these conventional carcinomas by dedifferentiation or metaplasia [4]; however, their origin is still debatable.

Comprehensive molecular profiling may provide genomic drivers of tumorigenesis to help elucidate the origin and characteristics of this rare neoplasm. A comprehensive view of the molecular landscape of gallbladder cancer has been provided using molecular profiling in specimens with adenocarcinoma and adenosquamous histologies [5]. Aneuploidy has been found in undifferentiated carcinomas of the gallbladder [3]. However, the genetic mutations in undifferentiated carcinoma cells remain unknown. Furthermore, there is lack of genetic studies on adjacent foci conventional cancers in comparison with undifferentiated carcinomas to elucidate the differences between them. In this report, we present a case of undifferentiated carcinoma of the gallbladder. In addition to standard pathological tests, we performed targeted gene sequencing on both the undifferentiated carcinoma and the foci adenocarcinoma for the first time to gain insight into their pathogenesis.

## Case presentation

**Fig. 1:**
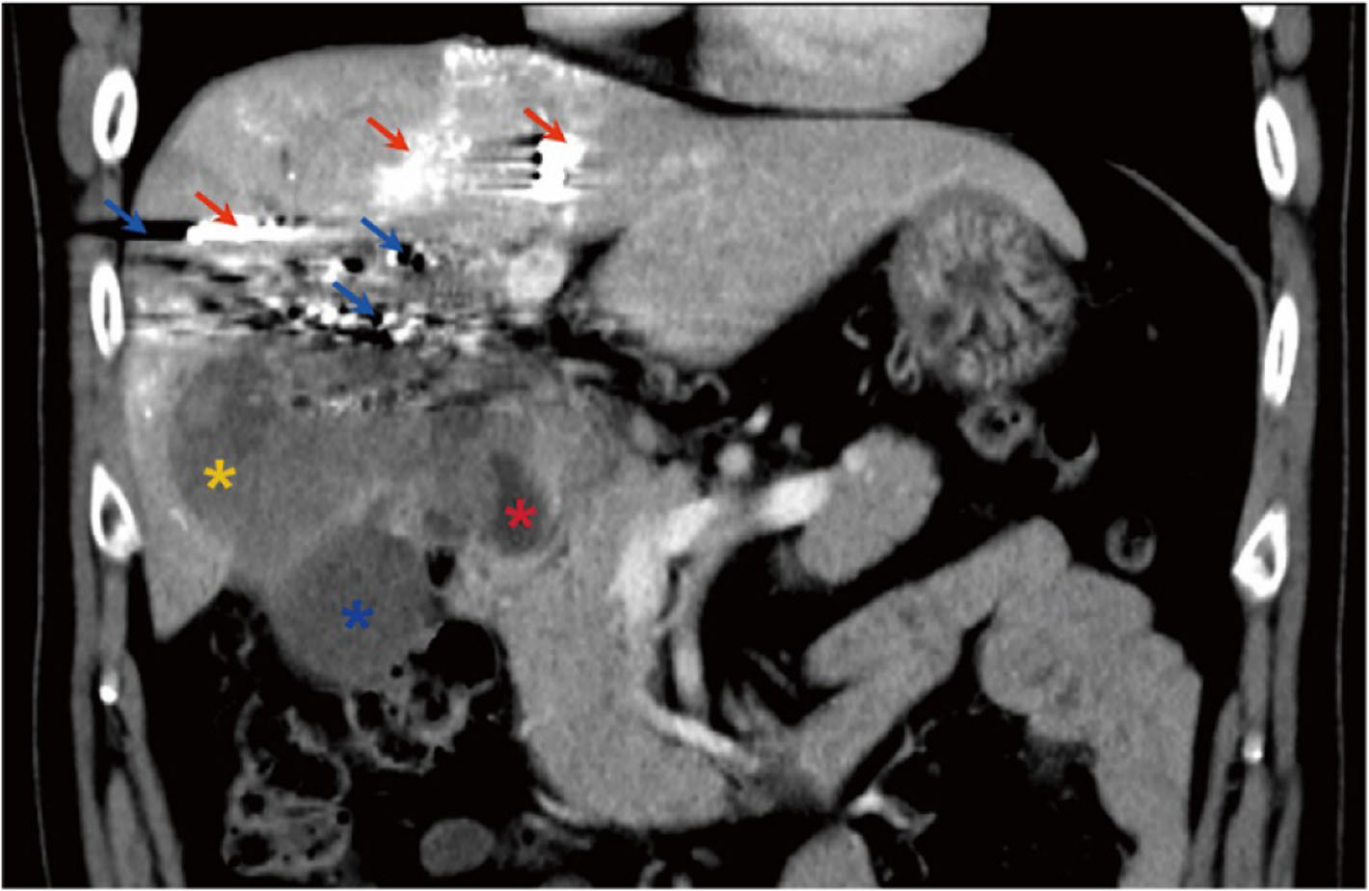
CT scan showing the mass located in the gallbladder, liver, and duodenum. The blue asterisk represents the non-tumor fundus of the gallbladder. The yellow asterisk represents the liver parenchyma infiltrated by the tumor. The red asterisk represents the tumor protruding into the duodenal lumen. The red arrows represents iodipin on CT images. The blue arrows represents CT artifacts

A 56-year-old man presented with upper abdominal pain, jaundice of the skin and sclera, itching, and an unexplained weight loss of about 15 pounds in 1 year. He was afebrile and had no nausea, vomiting, diarrhea, or other discomforts. Physical examination revealed upper abdominal tenderness and a palpable mass. Laboratory studies showed an elevated CA 19–9 level of 136.75 U/mL (upper limit of the normal range: 37 U/mL). He had a medical history of tuberculosis and hypertension, which had been well controlled with medications. An abdominal computer tomography (CT) scan revealed an 8 × 7 × 5- cm solid mass in the gallbladder, which had infiltrated the hilar bile duct, adjacent liver parenchyma, and duodenum (Fig. 1). The patient underwent hepato-pancreaticoduodenectomy combined with portal vein reconstruction 3 weeks after percutaneous transhepatic portal vein embolization.

**Fig. 2:**
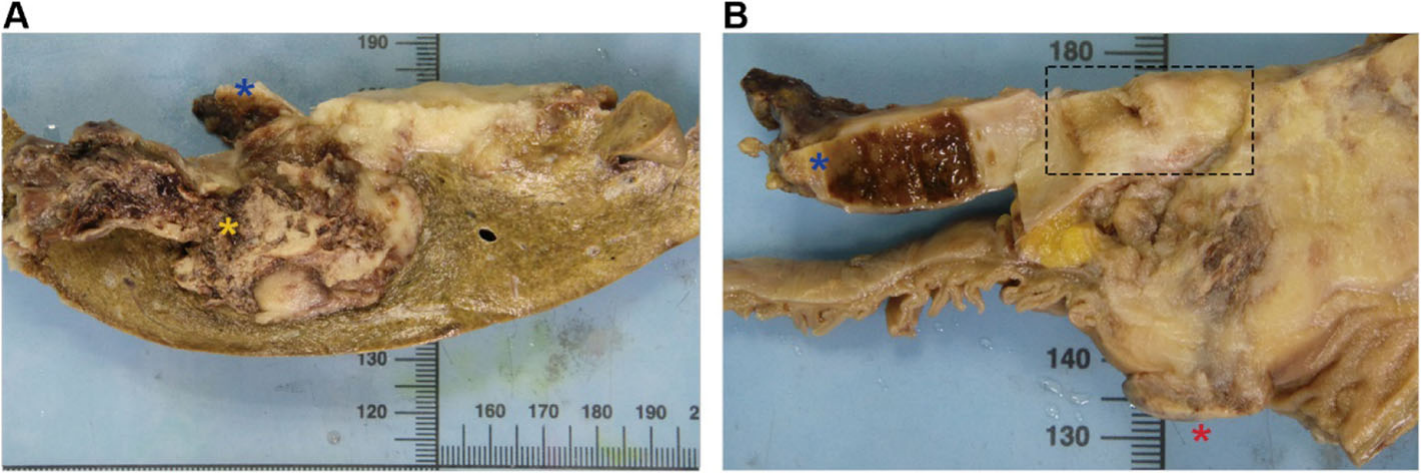
Gross appearance of the mass in the cut surface of the resected specimen. **a** The cut surface shows the mass perforating the gallbladder wall, with invasion into the liver parenchyma. **b** The cut surface shows invasion of the mass into the duodenal wall and protrusion into the duodenal lumen. The foci of the mass in the neck of the gallbladder (black dotted box) has a papillary appearance. The blue asterisk represents the non-tumor fundus of the gallbladder. The yellow asterisk represents the liver parenchyma infiltrated by the tumor. The red asterisk represents the tumor protruding into the duodenal lumen. The black dotted box highlights the focal papillary tumor

Grossly, a hard 8 × 6 × 6-cm solid mass occupied the neck and body of the gallbladder in continuity with the mucosal surface of the fundus (Fig. 2a–b). The mass had perforated the serosa of the gallbladder wall, with direct invasion into the adjacent organs including the liver (with a size of 10 × 9 × 8 cm) (Fig. 2a), duodenum (invading the whole layer of the duodenal wall and forming a 3-cm in diameter nodule, protruding into the duodenal lumen) (Fig. 2b), and the serosa of the stomach. The cut surface of the mass in the gallbladder was gray-white, relatively soft, and showed apparent hemorrhage in the liver portion (Fig. 2a). A focal papillary tumor was present in the neck of the gallbladder (Fig. 2b).

**Fig. 3:**
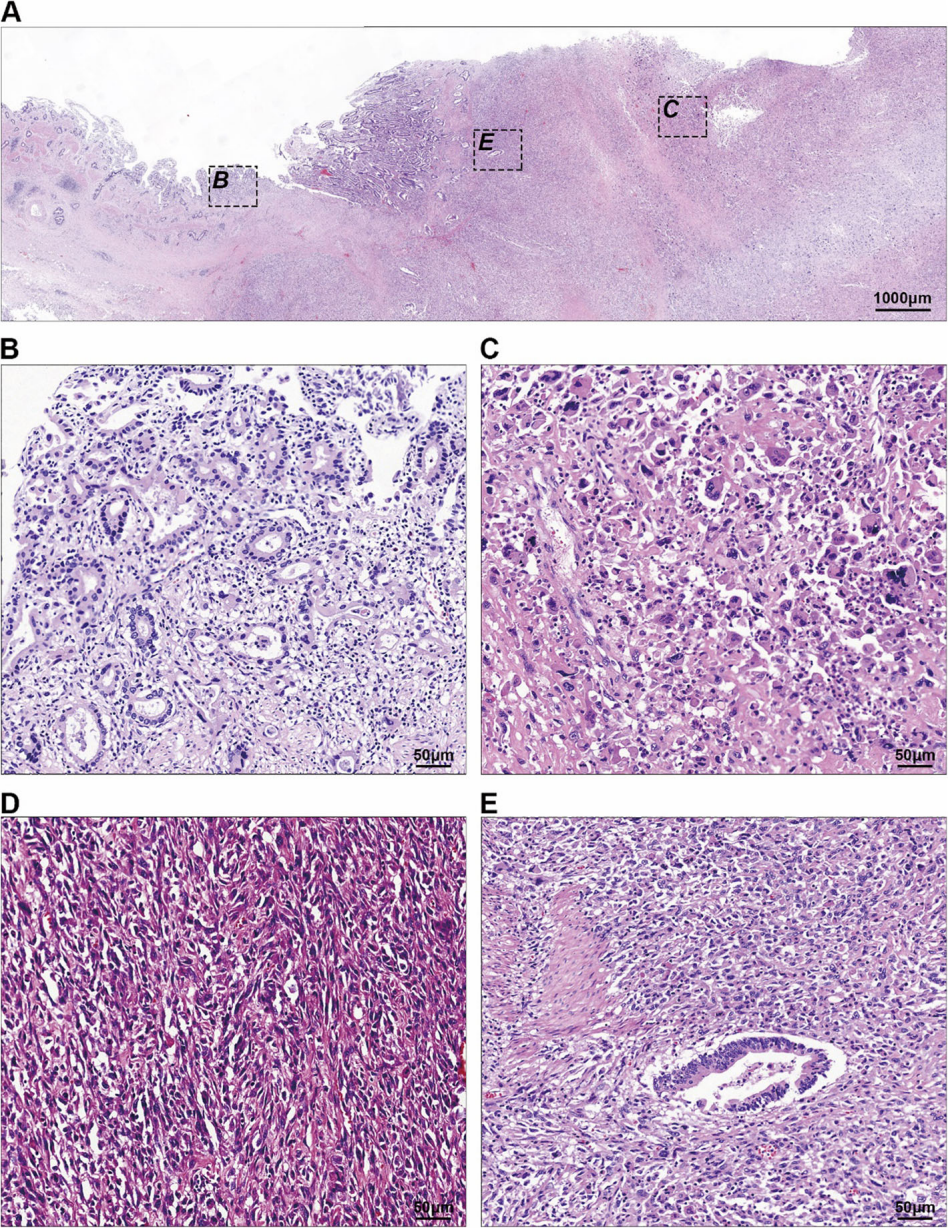
Histological appearance of the undifferentiated carcinoma after hematoxylin and eosin (H&E) staining. **a** Whole-slice scan of the corresponding black dotted box in Fig. 2b. **b** Tubular pattern of the tumor corresponding to the “***b***” black dotted box in Fig. 3a. **c** Undifferentiated pattern with pleomorphic mononuclear cells of the tumor corresponding to the “***c***” black dotted box in Fig. 3a. **d** Undifferentiated pattern with spindle cells of the tumor (not included in Fig. 3a). **e** The transition part of the tumor corresponding to the “***e***” black dotted box in Fig. 3a. Original magnification: × 10 (**a**); × 200 (**b-d**)

**Fig. 4:**
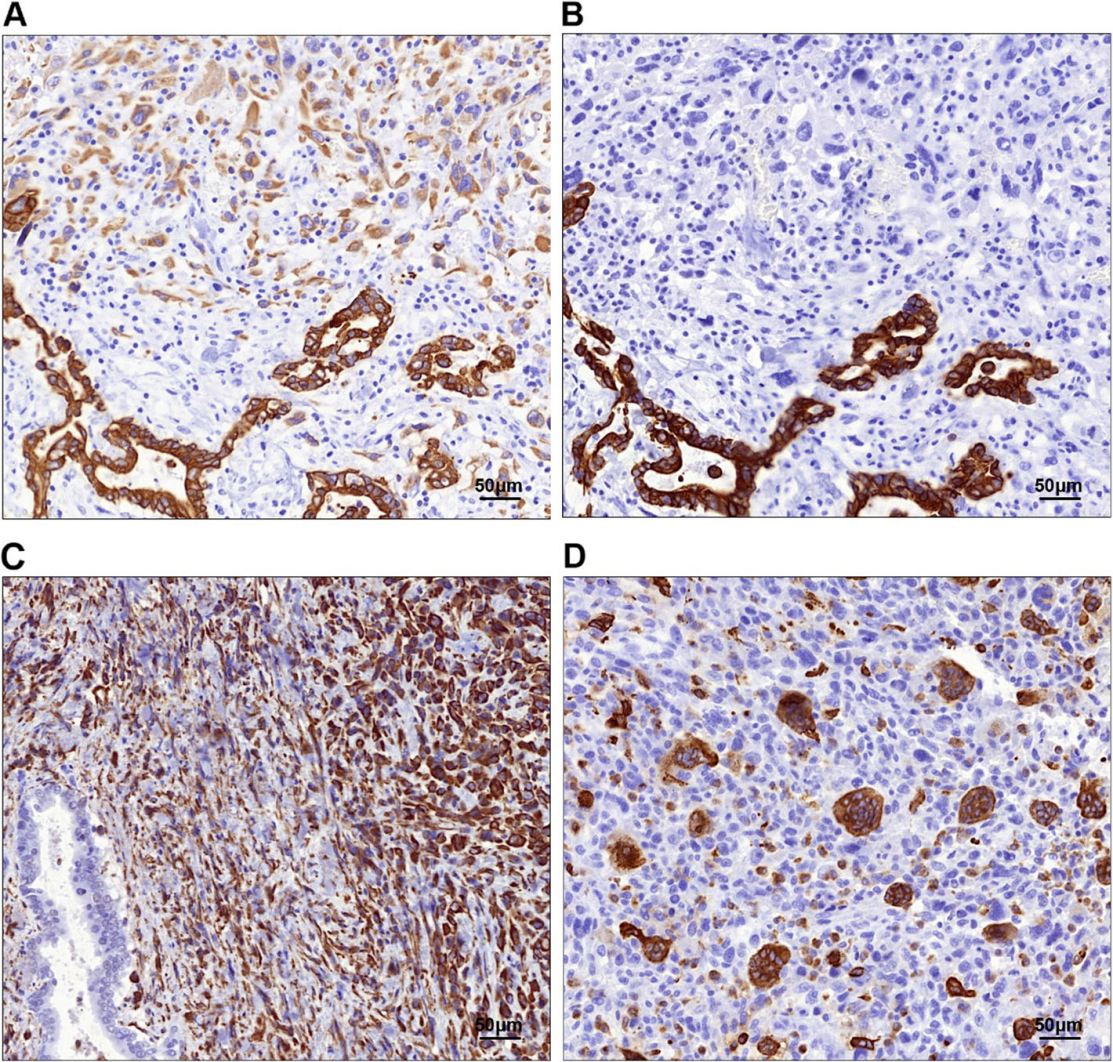
IHC findings of the undifferentiated carcinoma. **a** Cells in both the tubular and undifferentiated components were stained for AE1/AE3. **b** Positive immunostaining for CK7 was identified only in the cells of the tubular component. **c** The cells of the undifferentiated component were “diffuse positive” for Vimentin. **d** The osteoclast-like giant cells were strongly positive for CD68. Original magnification: × 200

Histologically, the tumor was characterized by two different architectural patterns (Fig. 3a). The first pattern was a well-differentiated tubular adenocarcinoma (Fig. 3b), which corresponded to the papillary tumor on gross findings and occupied < 5% of the entire tumor. This component was mainly confined to the local mucosal layer but had partially invaded the muscle layer of the gallbladder. The second pattern, which was the predominant component, was composed of pleomorphic mononuclear cells or poorly cohesive spindle-shaped cells with diffuse distribution (Fig. 3c–d). The cells were densely packed and had eosinophilic cytoplasms, irregular nuclear shapes, coarse chromatins, numerous abnormal mitoses, and occasional hemosiderin deposits. Some mononuclear cells showed anaplasia with bizarre nuclei (Fig. 3c). Some spindle cells had broad cytoplasms and elongated nuclei that resembled rhabdomyosarcoma cells (Fig. 3d). Notably, the density of the spindle-shaped cells increased in areas distant from the tubular pattern. The spindle-cell density was greatest in the invasive portions of the tumor in the liver, duodenum, and stomach; it was accompanied by focal hemorrhage and necrosis. The two patterns were continuous, with a transition zone between them (Fig. 3e). The tubular component was strongly positive for the epithelial markers AE1/AE3 (Fig. 4a), CK7 (Fig. 4b) and CEA. The cells in the undifferentiated component were weakly diffusely positive for AE1/AE3 in the transition zone, weakly focally positive for AE1/AE3 in distant areas, and strongly diffusely positive for vimentin (Fig. 4c); they were consistently negative for neurocrine markers (SYN, CGA) and sarcoma-differentiated markers, including SMA, desmin, MyoD1, S100, DOG1, CD117, and CD31. The cells in both components were negative for P53. The proliferative index of Ki67 was higher in the undifferentiated component (40%) than in the tubular component (20%). In addition, osteoclast-like giant cells were occasionally dispersed among the tumor cells, and contained 5–30 uniform small ovoid nuclei in the center, with no anaplasia and mitosis. The osteoclast-like giant cells were positive for vimentin and the macrophage marker CD68 (Fig. 4d). Based on these findings, we made a pathological diagnosis of an undifferentiated carcinoma.

Tumor DNA from the undifferentiated and tubular components was tested for 448 cancer-related genes. Each component contained a minimum of 70% of tumor cells. Analyses of single nucleotide variant (SNV), insertion or deletion (INDEL), gene fusion, and copy number variation (CNV) were performed by next-generation sequencing (NGS) with NovaSeq 6000 (Illumina, San Diego, CA) using a custom-designed panel (Amoy Diagnostics, Xiamen, China). The panel covered all exons and selected introns of 448 cancer-related genes. As a result, four somatic mutations (*TP53* c.880G > T: p.E294*; *TERT* c.-124C > T [promoter mutation]; *ARID2* c.2278C > T: p.Q760*; and *CDH1* c.1477G > A: p.V493M) and three amplifications (*CCND1, FGF19,* and *MET*) were detected in the undifferentiated component. In contrast to these findings, the tubular component contained 102 somatic mutations (see Additional file 1) and one amplification, which included the four mutations and one amplification (*CCND1*) also found in the undifferentiated component. The details of shared gene mutations are listed in Table 1. In addition, the tumor mutation burden (TMB) was calculated as the total number of non-synonymous somatic mutations per megabase (MB) of genome. This patient had a TMB of 3.45 muts/Mb in the undifferentiated carcinoma and a considerably higher TMB of 87.07 muts/Mb in the adenocarcinoma.

**Table 1:** Shared gene alterations of the undifferentiated and tubular components in the undifferentiated gallbladder carcinoma

Number	Gene	Mutation	CNV^**a**^/ VAF^**b**^	
			Tubular	Undifferentiated
1	CCND1	Amplification	31.25	25.85
2	TP53	NM_000546.5: c.880G > T	16.30%	25.61%
3	TERT	NM_198253.2: c.-124C > T	24.73%	30.55%
4	ARID2	NM_152641.2: c.2278C > T	19.84%	28.10%
5	CDH1	NM_004360.3: c.1477G > A	12.78%	17.12%

^**a**^*CNV* Copy number variation; ^**b**^*VAF* Variation of allele frequency

The patient died of tumor recurrence in the residual liver and peritoneum 2 months after the surgery without adjuvant treatment.

## Discussion

This is the first report that separately describes the genetic alterations in the undifferentiated and tubular components of the undifferentiated gallbladder carcinoma. The tubular component in this tumor contained 102 gene mutations associated with adherens junction, cell cycling, apoptosis, DNA repair, epithelial–mesenchymal transformation, DNA methylation, and mitogen-activated protein kinase (*MAPK*) signaling. The undifferentiated component had only four gene mutations, which were also present in the adenocarcinomatous portion of the tumor. The shared gene alterations and continuous transition zones suggest that the two different components might have the same origin. Undifferentiated carcinomas occur more frequently in the pancreas and contain the same *KRAS* mutation as that in pancreatic ductal adenocarcinoma, suggesting that the two components have a common origin [6]. However, the mutations in this undifferentiated gallbladder carcinoma differ from those found in undifferentiated carcinomas of the pancreas or the extrahepatic bile ducts [6, 7]. These indicate that different components of the undifferentiated carcinoma may have the same origin and that the undifferentiated gallbladder carcinoma had unique molecular alterations.

In the present case, although the two tumor components had shared gene alterations and continuous transition zones, the TMB of the two components was quite different. TMB variation can be caused by tumor cell content and intratumor heterogeneity [8]. Intratumor heterogeneity and associated tumor branched evolution have been demonstrated in renal, lung, and liver carcinomas using multiregion sequencing [9–11]. Biliary tract cancers including gallbladder carcinoma have high molecular heterogeneity [12]. It is possible that the remarkably high TMB of the tubular component of this carcinoma was due to intratumor heterogeneity and that the heterogeneity-associated branched evolution was responsible for the development of the undifferentiated carcinoma. The subclone of undifferentiated components contained *FGF19* and *MET* amplifications that is not found in the tubular component and is reported to be associated with poor prognosis in liver and gallbladder carcinomas [13, 14]. The two genetic amplifications may contribute to tumor progression toward more invasiveness and worse prognosis.

Our patient relapsed and died 2 months after the operation without adjuvant treatment. The poor outcome illustrates the aggressive behavior of this tumor, consistent with the previously reported cases [15]. This suggests that surgery alone may not be sufficient, and thus multidisciplinary adjuvant therapy should be investigated to improve the survival of patients with advanced undifferentiated gallbladder carcinomas.

In conclusion, this case report provides new insights into the molecular genetic features of undifferentiated carcinomas. It is the first report to describe the similarities and differences in genetic alterations between the undifferentiated carcinoma and the foci adenocarcinoma. The similarities between them indicate a common origin. The differences support that heterogeneity-associated branched evolution of the adenocarcinoma may contribute to the occurrence of undifferentiated carcinomas. However, further studies in more cases are required.

## Supplementary information

**Additional file 1.** The 102 somatic mutations and corresponding signaling pathway in the tubular carcinoma component of the gallbladder carcinoma.

## Data Availability

The most datasets during the study are included within the article.

## Abbreviations

CT: Computer tomography
SNV: Single nucleotide variant
INDEL: Insertion or deletion
CNV: Gene fusion and copy number variation
NGS: Next-generation sequencing
